# FF-ViT: probe orientation regression for robot-assisted endomicroscopy tissue scanning

**DOI:** 10.1007/s11548-024-03113-2

**Published:** 2024-04-10

**Authors:** Chi Xu, Alfie Roddan, Haozheng Xu, Giannarou Stamatia

**Affiliations:** Hamlyn Centre for Robotic Surgery Department of Surgery and Cancer, Imperial College London, London, SW7 2AZ UK

**Keywords:** Fast Fourier transform, Transformer, Cross-attention, Endomicroscopy

## Abstract

**Purpose:**

Probe-based confocal laser endomicroscopy (pCLE) enables visualization of cellular tissue morphology during surgical procedures. To capture high-quality pCLE images during tissue scanning, it is important to maintain close contact between the probe and the tissue, while also keeping the probe perpendicular to the tissue surface. Existing robotic pCLE tissue scanning systems, which rely on macroscopic vision, struggle to accurately place the probe at the optimal position on the tissue surface. As a result, the need arises for regression of longitudinal distance and orientation via endomicroscopic vision.

**Method:**

This paper introduces a novel method for automatically regressing the orientation between a pCLE probe and the tissue surface during robotic scanning, utilizing the fast Fourier vision transformer (FF-ViT) to extract local frequency representations and use them for probe orientation regression. Additionally, the FF-ViT incorporates a blur mapping attention (BMA) module to refine latent representations, which is combined with the pyramid angle regressor (PAR) to precisely estimate probe orientation.

**Result:**

A first of its kind dataset for pCLE probe-tissue orientation (pCLE-PTO) has been created. The performance evaluation demonstrates that our proposed network surpasses other top regression networks in accuracy, stability, and generalizability, while maintaining low computational complexity (1.8G FLOPs) and high inference speed (90 fps).

**Conclusion:**

The performance evaluation study verifies the clinical value of the proposed framework and its potential to be integrated into surgical robotic platforms for intraoperative tissue scanning.

**Supplementary Information:**

The online version contains supplementary material available at 10.1007/s11548-024-03113-2.

## Introduction

Through advances in biophotonics, pCLE technology has been developed, which has made it possible to directly examine tissue at a microscopic scale during open surgery. Preliminary studies have shown that pCLE can help identify residual cancer tissue, leading to higher rates of tumor removal [1, 2]. To obtain high-quality pCLE data, it is necessary to keep the probe at an optimal working distance of micrometer scale [3, 4]and perpendicular to the tissue surface [5, 6].

To increase scanning precision, visual servoing methods based on microscopic vision have recently been proposed using blur metrics with statistical analysis methods [3] and deep learning regression methods [4], to refine the distance between the probe and the tissue surface (probe-tissue distance). These robotic pCLE scanning methods are limited to only controlling the longitudinal distance between the probe and the tissue, maintaining the pCLE probe within the optimal scanning distance. However, it is equally important to ensure that the probe is always oriented perpendicular to the tissue surface. This is vital for the acquisition of high-quality pCLE data, particularly when dealing with uneven tissue surfaces, such as the cavities after tumor resection.

To control the orientation of the pCLE probe, robotic pCLE tissue scanning methods have been proposed using force sensors or macroscopic vision. Wisanuvej et al. [5] designed a micro-scanning robotic tool and utilized force sensor-based feedback control to refine the orientation of the pCLE probe. However, during surgery, the force sensor-based approach needs to be calibrated for different types of tissues, which is time-consuming. Zhang et al. [6] reconstructed the 3D map of the tissue surface and applied maker-based probe pose estimation to infer the orientation of the probe with respect to the tissue surface during scanning. However, this method is only effective when the tissue is not deforming, and its accuracy can be affected by the surgical environment’s lighting conditions [7–9]. More importantly, the error of 3D construction approach is of millimeter scale (endomicroscopy requires micrometer scale), which causes large probe-tissue orientation error.

In this paper, we introduce the first microscopic vision method for automatic inference of the orientation of the pCLE probe with respect to the normal vector of the tissue surface during robotic tissue scanning.^1^ Our method leverages the patch-patch learning (self-attention) approach from the vision transformer (ViT) [10, 11] and patch-blur learning (cross-attention) approach from a BMA module to learn the probe orientation by analyzing the blurriness difference between image patches, and the correlation between image patch representations and blurriness (patch-blur correlation). To achieve this, we introduce the following contributions:

1. A fast Fourier patching embedding (FF-PE) layer is proposed, which divides input images into patches and learns data representations in the frequency domain.
2. A novel Pyramid Angle Regressor (PAR) which includes a blur mapping attention (BMA) module is developed (denoted as PAR-BMA) to incorporate blurriness information into the data representation for better predictions.

**Fig. 1:**
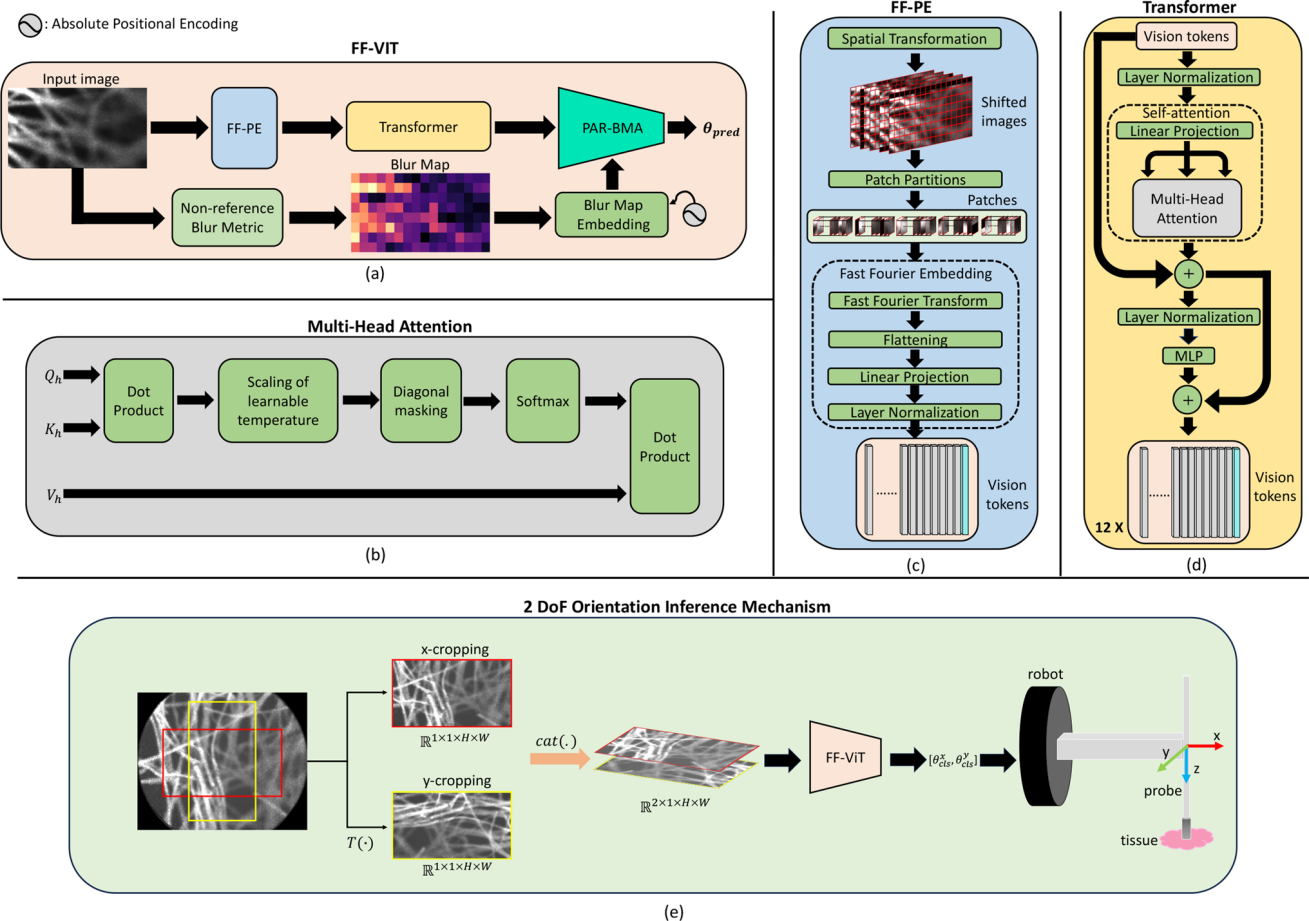
**a** The overall architecture of FF-ViT. **b** The multi-head attention module. **c** and **d** The inner structures of FF-PE and transformer, respectively. **e** The framework of 2 DOF orientation inference mechanism for robotic controlling

In order to overcome the lack of available training data, a microsurgical robot [12] was used to collect the first pCLE-PTO dataset, which consists of ex-vivo images and the corresponding probe orientations with respect to the tissue surface normal. Our performance evaluation confirms that our proposed network outperforms other SOTA regression networks in terms of accuracy, stability and generalizability. Our probe orientation regression model can be combined with any longitudinal distance regression model [4, 13–16] to control the pose of the pCLE probe for optimal robotic tissue scanning.

## Methods

In this work, we introduce the FF-ViT model which estimates the pCLE probe orientation during tissue scanning, by analyzing the proportion and location of blurry regions in the image, as well as the degree of blurriness in these regions. FF-ViT is composed of the FF-PE layer and the PAR-BMA module which learn image blur in the frequency domain and patch-blur correlation, respectively. The overall architecture of the FF-ViT is depicted in Fig. 1a.

First, a pCLE image $I_\mathrm{{in}}\in {\mathbb {R}}^{1\times H \times W}$ is inputted into the FF-ViT model, where the FF-PE layer $e(\cdot , \phi _\mathrm{{e}})$ is applied to spatially shift and split the image into patches. These patches are then converted to the frequency domain, and they are extracted as vision tokens ${\textbf{T}}_\mathrm{{e}} = e(I_\mathrm{{in}}, \phi _\mathrm{{e}})$, where $\phi _\mathrm{{e}}$ represents the learnable parameters of the FF-PE layer, and ${\textbf{T}}_\mathrm{{e}}=[{\textbf{T}}_\mathrm{{patch}};{\textbf{T}}_\mathrm{{cls}}] \in {\mathbb {R}}^{(N_\mathrm{{p}} + 1)\times C}$, with $N_\mathrm{{p}} + 1$ equal to the number of patches plus the *cls* token, and *C* represents the number of dimensions of the tokens. The transformer $f_\mathrm{{t}}(\cdot , \phi _t)$ then takes the vision tokens as input and learns the correlations between patches to enhance the data representation in ${\textbf{T}}_\mathrm{{t}} = f_\mathrm{{t}}({\textbf{T}}_\mathrm{{e}},\phi _\mathrm{{t}})$, where $\phi _\mathrm{{t}}$ are the learnable parameters of the transformer, and ${\textbf{T}}_\mathrm{{t}} \in {\mathbb {R}}^{(N_\mathrm{{p}} + 1)\times C}$. Finally, the PAR-BMA module $p(\cdot , \phi _\mathrm{{p}})$ takes the ${\textbf{T}}_\mathrm{{t}}$ and the embedded blur map ${\textbf{M}}_\mathrm{{e}} \in {\mathbb {R}}^{N_\mathrm{{p}}\times C}$, which is generated by a non-reference blur metric method [17], as inputs to predict $\varvec{\theta _\mathrm{{pred}}} = p({\textbf{T}}_\mathrm{{t}}, {\textbf{M}}_\mathrm{{e}}, \phi _\mathrm{{p}})$, where $\phi _\mathrm{{p}}$ is the learnable parameters of PAR-BMA, and $\varvec{\theta _\mathrm{{pred}}}$ is the set of predicted orientations obtained from multi-scale vision tokens.

### FF-PE layer

The FF-PE layer $e(\cdot , \phi _\mathrm{{e}})$ consists of three components, namely the spatial transformation, patch partition and fast Fourier embedding. Details of this layer are depicted in the blue box of Fig. 1c. The input of the FF-PE layer is $I_\mathrm{{in}}$, and a spatial transformation is used to shift $I_\mathrm{{in}}$ vertically and horizontally by a fixed number of pixels. This generates an augmented image $I^a_\mathrm{{in}} \in {\mathbb {R}}^{5\times H\times W}$, which contains four shifted images and the original input image $I_\mathrm{{in}}$. Then, the patch partition is used to split $I^a_\mathrm{{in}}$ into patches ${\textbf{P}} \in {\mathbb {R}}^{N_\mathrm{{p}} \times 5 \times H_\mathrm{{p}} \times W_\mathrm{{p}}}$, where $H_\mathrm{{p}}$ and $W_\mathrm{{p}}$ represent the height and width of patches, respectively. $N_\mathrm{{p}}$ is the number of patches ($N_\mathrm{{p}} = \frac{H}{H_\mathrm{{p}}} \cdot \frac{W}{W_\mathrm{{p}}}$).

In the frequency domain, blurry images are mainly represented by low-frequency signals, while clear images by high-frequency signals. To utilize this correlation between blurriness and low-frequency signals, the redundancy-free fast Fourier transform ${\mathcal {F}}(\cdot )$ [4, 18, 19] is used to convert the image patches to the frequency domain ${\mathcal {F}}({\textbf{P}}) \in {\mathbb {Z}}^{N_\mathrm{{p}}\times 5\times H_\mathrm{{p}} \times (\frac{W_\mathrm{{p}}}{2} +1)}$. The real and imaginary parts of ${\mathcal {F}}({\textbf{P}})$ are concatenated along the second dimension, resulting in ${\mathbb {R}}^{N_\mathrm{{p}}\times 10\times H_\mathrm{{p}} \times (\frac{W_\mathrm{{p}}}{2} +1)}$. To extract features from each patch, ${\mathcal {F}}({\textbf{P}})$ is flattened and projected into the vision tokens via layer normalization and linear projection. A learnable $\textit{cls}$ token sampled from a normal distribution ${\mathcal {N}}(0, {\textbf{I}})$ is concatenated to the above set of tokens.

### Transformer

In FF-ViT, the transformer consists of 12 attention-based blocks, as shown in Fig. 1d. Each block is composed of a self-attention layer, a multilayer perceptron (MLP), and layer normalization. Once the vision token ${\textbf{T}}_\mathrm{{e}}$ is generated by the FF-PE layer, a learnable position encoding vector $v_\mathrm{{p}}\in {\mathbb {R}}^C$ is added to each token $\alpha$ as ${\textbf{T}}^{\alpha }_\mathrm{{e}} = {\textbf{T}}^{\alpha }_\mathrm{{e}} + v_\mathrm{{p}}$, where $v_\mathrm{{p}}$ is sampled from a normal distribution ${\mathcal {N}}(0, {\textbf{I}})$. Then, ${\textbf{T}}_\mathrm{{e}}$ enters the sequence of attention-based blocks. In each block, the self-attention layer projects ${\textbf{T}}_\mathrm{{e}}$ into three components, namely query *Q*, key *K* and value *V* as:

1 $$\begin{aligned} \begin{aligned} \left[ Q, K, V\right] = {\textbf{T}}_\mathrm{{e}} \cdot W + b, \end{aligned} \end{aligned}$$

where *Q*, *K* and $V \in {\mathbb {R}}^{(N_\mathrm{{p}}+1)\times C}$. $W \in {\mathbb {R}}^{C\times 3C}$ and $b\in {\mathbb {R}}^{3C}$ are the trainable weight matrix and bias vector of the linear projection, respectively. *Q*, *K* and *V* are split into $N_\mathrm{{h}}$ parts by the multi-head attention module (*i*.*e*.,  $Q_\mathrm{{h}}, K_\mathrm{{h}}, V_\mathrm{{h}} \in {\mathbb {R}}^{N_\mathrm{{h}}\times (N_\mathrm{{p}} +1)\times C_\mathrm{{h}}}$). The inner structure of the multi-head attention is depicted in Fig. 1b.

**Fig. 2:**
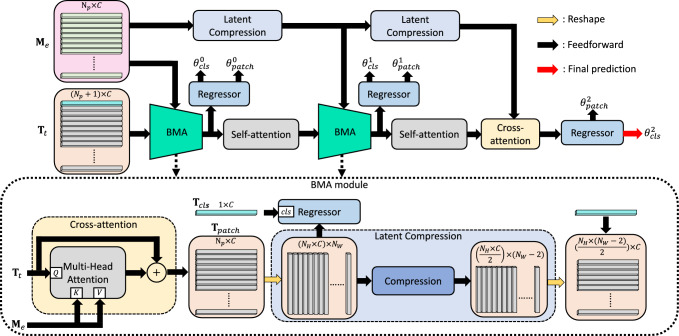
The architecture of PAR-BMA module and the BMA module

In the multi-head attention module, $Q_\mathrm{{h}}$ and $V_\mathrm{{h}}$ are multiplied to produce the *Q*-*V* attention map $A_\mathrm{{h}}\in {\mathbb {R}}^{N_\mathrm{{h}}\times (N_\mathrm{{p}} +1)\times (N_\mathrm{{p}} +1)}$. During the softmax operation, to eliminate the impact of self-token relations (diagonal elements of $A_\mathrm{{h}}$) against the inner-token relations (non-diagonal elements of $A_\mathrm{{h}}$), diagonal masking $\textrm{R}^{M}(\cdot )$ is applied to the attention map. Also, to enable the multi-head attention to learn the softmax temperature, the learnable temperature scaling $\tau$ is applied [11]. This is estimated by normalizing $A_\mathrm{{h}}$ to produce $\hat{A_\mathrm{{h}}}$ as follows:

2 $$\begin{aligned} \text {R}_{i, j}^{M}({\textbf{x}})=\left\{ \begin{array}{ll}\text {R}_{i, j}({\textbf{x}}) &  (i \ne j) \\ -\infty &  (i=j)\end{array}\right. , \end{aligned}$$

3 $$\begin{aligned} {\hat{A}}_{h} = \text {softmax}\left( \frac{\text {R}^{M}(A_\mathrm{{h}})}{\tau }\right) , \end{aligned}$$

where *i* and *j* are the row and column index, respectively, and ${\textbf{x}}$ is a square matrix. Equation (2) sets all the diagonal elements of $A_\mathrm{{h}}$ to $-\infty$. The resulting multi-head vision tokens ${\textbf{T}}_\mathrm{{h}}$, which contain only inner-token relations, are obtained by the dot product between $\hat{A_\mathrm{{h}}}$ and $V_\mathrm{{h}}$. Then, they are reshaped back to the size of ${\textbf{T}}_\mathrm{{e}}$. These vision tokens are then analyzed by the MLP with layer normalization to extract the desired features. This attention-based block is performed 12 times in the transformer to produce the vision tokens ${\textbf{T}}_\mathrm{{t}}$.

### PAR-BMA module

As illustrated in Fig. 1a, the non-reference blur metric method [17] is applied to the input image $I_\mathrm{{in}}$ to generate a blur map ${\textbf{M}}\in {\mathbb {R}}^{1\times N_\mathrm{{h}}\times N_W}$, where $N_\mathrm{{h}}$ and $N_W$ represent the height and width of the blur map, respectively. Then, the absolute positional encoding method [20] is applied to embed each blurry value of ${\textbf{M}}$, which is then reshaped to size $N_\mathrm{{p}} \times C$. The PAR-BMA module, depicted in Fig. 2, takes the outputs of the transformer ${\textbf{T}}_\mathrm{{t}}$ (light orange box) and the embedded blur map ${\textbf{M}}_\mathrm{{e}}$ (pink box) as inputs.

The PAR-BMA module consists of BMA layers, self-attention layers, latent compression modules and regressors. The BMA layer utilizes the cross-attention layer to learn the token-blur relations. Specifically, it projects ${\textbf{T}}_\mathrm{{t}}$ to *Q* and ${\textbf{M}}_\mathrm{{e}}$ to *K* and *V*. Following the cross-attention, the *cls* token ${\textbf{T}}_\mathrm{{cls}}$ is separated from ${\textbf{T}}_\mathrm{{t}}$, and the remaining patch tokens ${\textbf{T}}_\mathrm{{patch}}$ are fed into the latent compression (LC) module (light blue box in Fig. 2). LC is used to down-sample and summarize the latent representations. The LC module employs a compression technique, which involves a 1D average pooling layer with a kernel size of 3 and a stride of 1, followed by a 1D convolution layer with a kernel size of 1 and a stride of 1. These operations reduce the width of the latent representations by 2 and halve the height of the latent representations. Then, the compressed latent representations are shaped back to size $(\frac{N_\mathrm{{h}}\times (N_W -2)}{2})\times C$, and the ${\textbf{T}}_\mathrm{{cls}}$ is concatenated back to the compressed ${\textbf{T}}_\mathrm{{patch}}$. To enable the ${\textbf{T}}_\mathrm{{cls}}$ to learn information from the compressed ${\textbf{T}}_\mathrm{{patch}}$, the self-attention layer is applied to the recombined vision tokens. Overall, the ${\textbf{T}}_\mathrm{{t}}$ and the ${\textbf{M}}_\mathrm{{e}}$ are processed by the BMA module followed by the self-attention layers and the LC modules, to predict multi-scale probe orientations $\varvec{\theta _{pred}} = \left[ \theta ^0_\mathrm{{patch}},\theta ^0_\mathrm{{cls}}, \theta ^1_\mathrm{{patch}}, \theta ^1_\mathrm{{cls}}, \theta ^2_\mathrm{{patch}},\theta ^2_\mathrm{{cls}}\right]$. As shown in Fig. 2, $\theta ^2_\mathrm{{cls}}$ is selected as the final probe orientation as this is the results of the last inference.

### Loss function

To ensure that the network learns the correlation between orientation and blurriness at various scales of latent representation, the $L_1$ loss is applied to the predicted probe orientation $\varvec{\theta _{pred}}$ at multiple scales,

4 $$\begin{aligned} {\textbf{L}} = \frac{1}{2N}\sum _{m\in \left[ cls, patch\right] }\sum ^{N}_{n=0} \gamma ^{n}\cdot \left\| \theta _{GT} - \theta ^n_m\right\| , \end{aligned}$$

where *N* is the number of scales and $\theta _{GT}$ is the ground truth probe orientation with respect to the normal vector of the tissue surface. The $\gamma$ is the decay coefficient, which is set to 0.5.

### 2 DoF orientation inference

For pCLE orientation control, rotations along only x- and y-axis affect the orientation of the pCLE probe with respect to the tissue surface, as shown in Fig. 1e. For the pCLE imaging system, any deviation of the probe orientation from the normal vector of the tissue surface in the *x*-axis can induce notable difference in the blurriness between the left and right areas of the pCLE image. Similarly, rotation along the *y*-axis will create significant difference in the blurriness of the top and bottom image areas. As detailed in Sect. 2, the proposed approach can regress a probe-tissue orientation by analyzing the distribution of blurry regions (e.g., left–right blurriness discrepancies) in the input pCLE image $I_\mathrm{{in}}$. To achieve this, the *x*- and *y*-cropping operations (red and yellow borders, respectively, in Fig. 1e) are applied to the pCLE images, yielding the cropped images $I_x, I_y \in {\mathbb {R}}^{1\times 1\times H\times W}$ which are used to regress the probe orientation along the *x* and *y* rotation axis, respectively. To empower the model to regress orientations in *x* and *y* rotation axis, we have designed a 2 DoF orientation inference mechanism, illustrated in Fig. 1e. In the pCLE image, the areas within the red and yellow borders represent $I_x$ and $I_y$, respectively. Subsequently, $I_x$ and $I_y$ are concatenated along the batch dimension ${\mathbb {R}}^{2\times 1\times H\times W}$ and are then inputted into the FF-ViT to deduce the probe orientations relative to the tissue surface in the *x* and *y* dimensions [$\theta ^x_\mathrm{{cls}}$, $\theta ^y_\mathrm{{cls}}$]. To keep the probe always perpendicular to the tissue surface, the robot moves the probe according to the regressed [$\theta ^x_\mathrm{{cls}}$, $\theta ^y_\mathrm{{cls}}$]. This allows the probe to achieve optimal alignment relative to the tissue surface.

## Experiments and analysis

**Fig. 3:**
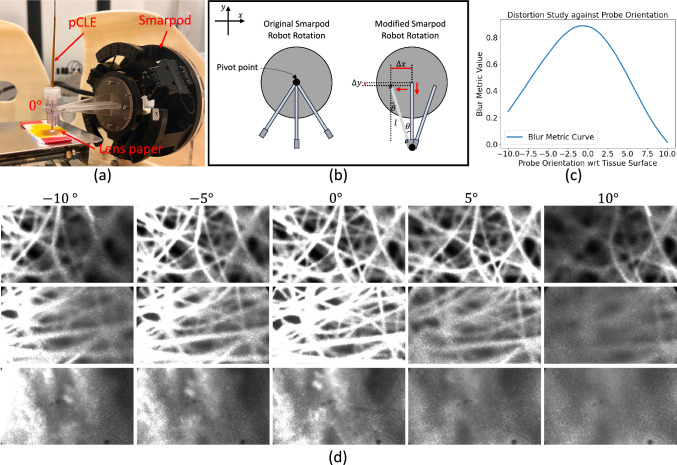
**a** pCLE-PTO dataset collection setup. **b** The pivot point rotation for tissue scanning. **c** The distortion study of pCLE image against probe-tissue orientations. **d** pCLE-PTO sample data: the first two rows and the last row are pCLE images of lens paper and heart tissue, respectively

### pCLE-PTO dataset generation

The pCLE data were collected using the Cellvizio system (Mauna Kea Technologies, Paris), by scanning lens paper and ex-vivo cow heart tissue. The samples were stained with 0.1% Acriflavin. The Smarpod microsurgical robot (SmarAct, Germany) was used to control the miniprobe. As shown on the left side of Fig. 3b, the pivot point of the original Smarpod robot is at the base (center) of the robot. This moves the tip of the probe to different locations on the tissue during rotation instead of correcting its orientation at the same tissue spot.

To overcome this limitation, we implemented additional translations in the *x* and *y* dimensions ($\Delta x$ and $\Delta y$), which create different rotation angles $\theta$. This effectively shifts the pivot point to the tip of the miniprobe. We calculated the translations $\Delta x$ and $\Delta y$ as follows:

5 $$\begin{aligned} \Delta x = - l\cdot \sin {\theta }, \end{aligned}$$

6 $$\begin{aligned} \Delta y = l\cdot (\cos {\theta } - 1), \end{aligned}$$

where $\theta$ is the rotation angle and *l* refers to the distance from the base of the Smarpod robot to the tip of the miniprobe.

Following this modification, we applied the adjusted Smarpod robot rotation to collect pCLE data within a range of $-10^{\circ }$ to $10^{\circ }$ relative to the normal of the object’s surface, taking steps of $0.5^{\circ }$, as shown in Fig. 3a.

Our data collection yielded a total of 255 lens paper videos and 32 cow heart videos, each associated with specific probe-tissue orientations. We used 239 lens paper videos (9389 frames) for training, while 26 lens paper videos (1066 frames) and 32 cow heart videos (1312 frames) were allocated for testing. Figure 3d displays sample images from our dataset.

To determine the working range for the probe orientation which generates pCLE images with minimal blur, we conducted a distortion study by measuring the image blur with respect to the probe orientations using blur metrics (MoI, NFBM, H-FFT, LAPV, GEDR as included in the Blur Metrics section of Supplementary Material), as presented in Fig. 3c. The distortion rate between adjacent orientations was calculated as the difference in their corresponding blur metric values. Within the range from $-1.5^{\circ }$ to $1.5^{\circ }$, the average distortion rate was 1.1%, while outside this range, the distortion rate increased by a factor of four (4.3%). As such, we define the working range for the probe orientation as [$-1.5^{\circ }$,$1.5^{\circ }$]. This finding helps to establish a standard for optimal probe orientation during scanning procedures.

### Model implementation

The models were developed using the PyTorch [21] library and run on a NVIDIA RTX A5000 with 24GB memory. The input image size was set to $256\times 488$, and the patch size for FF-ViT was $32 \times 28$. In the cross-attention layer, the number of pixels to shift for the spatial transformation was set to 1. In the BMA module, the diagonal masking is removed. In the FF-ViT, the transformer’s hidden dimension, depth, heads, and hidden dimension of MLP were set to 192, 12, 3, and 768, respectively. The training employed the AdamW optimizer [22] with weight decay of 0.01, a batch size of 512, and a learning rate of 0.0001.

### Comparison study

We compared our proposed method with SFFC-Net [4], a regression model for pCLE, as well as, with state-of-the-art (SOTA) deep learning frameworks for general regression tasks [11, 23–26]. To ensure a fair comparison, we trained all models with a fixed random seed. The models were exclusively trained on the lens paper training dataset. Their performance was tested on the lens paper and cow heart tissue test datasets, using the evaluation metrics of mean absolute error (**MAE**), standard deviation ($\varvec{\sigma }$), and rotation direction accuracy ($\varvec{Acc_{dir}}$). In experiments, each table highlights the best performance achieved in the corresponding evaluation metrics through bold values.

Table 1 presents the results of our comparison study between our proposed FF-ViT and other SOTA deep learning models. On the unseen lens paper dataset, our model has a slightly higher mean absolute error (**MAE**) than XCiT-T, Swin-T, ResNet18 and SFFC-Net. However, our model has a much lower standard deviation ($\varvec{\sigma }$) than these models. Furthermore, our model has the highest accuracy ($\varvec{Acc_{dir}}$). Therefore, our model’s prediction can guide the robot to approach the optimal probe orientation in the most stable way for high-quality pCLE images. The results on the cow heart data shown in Table 1 indicate that our model is minimally impacted when the data distribution changes from lens paper to cow heart compared to the other models. This finding verifies that our model is more generalizable than the others. Additionally, the spatial domain-based models (all the models except FF-ViT in Table 1) are more likely to be affected by switching the data distribution, than the frequency domain-based models (FF-ViT). This is because in that case, low-level information significantly changes in the spatial domain-based models.

**Table 1:** Comparison with SOTA models on the pCLE-PTO dataset

	Lens Paper			Cow heart		
Models	MAE	$\sigma$	Acc$_\mathrm{{dir}}$ (%)	MAE	$\sigma$	Acc$_\mathrm{{dir}}$ (%)
ResNet18 [23]	1.142	0.485	97.5	2.393	1.166	91.9
ConvNeXt [26]	1.486	0.628	94.7	2.722	1.404	84.1
SFFC-Net [4]	1.164	0.659	97.4	2.227	0.771	95.6
Swin-T [24]	1.143	0.473	96.2	2.795	0.781	90.5
XCiT-T [25]	**1**.**091**	0.590	95.8	2.121	0.961	89.1
ViT-SD [11]	1.562	0.706	95.5	2.507	0.883	92.4
FF-ViT	1.167	**0**.**469**	**98.5**	**1**.**549**	**0**.**336**	**97.0**

The MAE is the mean absolute error; $\sigma$ is the standard deviation of MAE; and Acc$_\mathrm{{dir}}$ is the rotation direction accuracy. The unit of MAE and $\sigma$ is $^\circ$

**Table 2:** Tenfold cross-validation

	Lens paper			Cow heart		
Models	MAE	$\sigma$	Acc$_\mathrm{{dir}}$ (%)	MAE	$\sigma$	Acc$_\mathrm{{dir}} (\%)$
ResNet18 [23]	1.162	0.573	94.0	2.451	0.981	89.9
SFFC-Net [4]	1.195	0.628	94.8	2.378	1.039	90.2
XCiT-T [25]	**1**.**158**	0.578	94.6	2.157	0.786	91.5
FF-ViT	**1**.**158**	**0**.**539**	**94.1**	**1**.**803**	**0**.**601**	**93.1**

**Table 3:** Ablation study

				Lens paper			Cow heart		
Models	FF-PE	PAR	BMA	MAE	$\sigma$	Acc$_\mathrm{{dir}}$ (%)	MAE	$\sigma$	Acc$_\mathrm{{dir}}$ (%)
ViT-SD		✔		1.521	0.522	95.1	2.426	0.827	92.6
ViT-SD		✔	✔	1.470	0.478	97.4	2.883	0.975	94.8
ConvNeXt		✔	✔	1.255	0.657	92.7	2.045	0.746	91.5
XCiT-T		✔	✔	**1**.**009**	0.467	97.2	1.959	0.797	92.3
FF-ViT	✔			1.366	0.548	95.3	2.755	1.531	91.3
FF-ViT	✔	✔		1.264	0.492	97.3	2.248	0.488	95.7
FF-ViT$^{\mathrm{w/o}}$	✔	✔	✔	1.183	0.522	95.6	2.103	0.691	95.0
FF-ViT	✔	✔	✔	1.167	**0**.**469**	**98.5**	**1**.**549**	**0**.**336**	**97.0**

The FF-ViT$^{\mathrm{w/o}}$ represents the FF-ViT without spatial transformation which is described in Sect. 2.1

### Tenfold cross-validation on pCLE-PTO dataset

In this study, to eliminate potential biases within the pCLE-PTO dataset, a tenfold cross-validation is conducted. In this experiment, the whole lens paper dataset is shuffled and split into 10 groups. Each unique group is combined with the unseen cow heart dataset to create the testing dataset, while the rest of the groups are used as the training dataset. For the tenfold cross-validation, we selected the best performing models, namely the SFFC-Net (regression model for pCLE image), ResNet18 (best performance CNN model in our comparison study), XCiT-T (best performance transformer model in our comparison study) and our proposed FF-ViT. The mean performance evaluation values of the tenfold cross-validation are shown in Table 2. The findings indicate that our FF-ViT model consistently outperformed the compared models on both datasets. Notably, on the heart dataset, the FF-ViT exhibited superior generalizability compared to the other models.

**Table 4:** Ablation study on the number of latent compression layers in the PAR-BMA module

	Lens Paper			Cow Heart		
Number of layers (*H* $\times$ *W*)	MAE	$\sigma$	Acc$_\mathrm{{dir}}$ (%)	MAE	$\sigma$	Acc$_\mathrm{{dir}}$ (%)
0 (8$\times$16)	**1**.**127**	0.494	94.4	2.09	0.694	92.3
1 (4$\times$14)	1.138	0.488	94.8	1.933	0.827	91.3
2 (2$\times$12)	1.167	0.469	**98.5**	**1**.**549**	**0**.**336**	**97.0**
3 (1$\times$10)	1.156	**0**.**430**	95.3	1.813	0.478	93.0

The *H*$\times$*W* is the size of the final feature map in the PAR-BMA module

### Ablation study

To analyze the individual contributions of each proposed component, an ablation study was conducted, and the results are presented in Table 3. The baseline model is the ViT-SD [11]. The ablation study shows that each component contributes positively to the overall architecture. As shown in Table 3, incorporating the FF-PE layer in the ViT-SD with the PAR/PAR-BMA module significantly improves the performance of the model on both datasets. This indicates that frequency features are more suitable and generalizable than spatial features for tasks related to image blur. The PAR module enhances the performance of ViT-SD and FF-ViT in terms of all evaluation metrics, demonstrating the effectiveness of supervising multi-scale latent representations during training. The contribution of the spatial transformation is also assessed at the last two rows of Table 3. On the cow heart dataset, the performance of the FF-ViT gets significant improvement by adding the spatial transformation, which means that it can improve the model’s generalizability.

To demonstrate the contribution of the PAR-BMA module, we also integrated it in our ablation study with two SOTA backbones namely, the ConvNeXt [26] and the XCiT-T [25]. The results in Table 3 reveal performance enhancement across both datasets for the ConvNeXt, XCiT-T, and FF-ViT models, with particularly notable improvement observed in the cow heart dataset. This underscores the PAR-BMA module’s substantial role in improving the models’ generalizability. Further exploration of the PAR-BMA module involved an ablation study focusing on the number of latent compression layers (scales), as presented in Table 4. This experiment established that the PAR-BMA configuration with two latent compression layers delivered superior performance on the cow heart dataset. Consequently, we adopted this two-layer structure for the latent compression layers in our proposed FF-ViT model.

### Inference time and computational cost

Our proposed model is designed to address a computer-assisted intervention (CAI) task, making the model’s inference time and computational cost critical performance indicators for real-time applications. This experiment was executed on a NVIDIA RTX A5000. To evaluate computational complexity, we assessed the model using three key metrics: floating-point operations (FLOPs), the number of parameters (Params) and the model’s inference time.

According to the experiment, the FF-ViT model exhibited a low count of FLOPs (1.8G) and Params (11.2M) and an inference time of 11 ms (90.1 fps), fitting the real-time applications well.

## Conclusion

In this paper, we have introduced the first framework to automatically regress the orientation of a pCLE probe with respect to the normal vector of the tissue surface for robotic tissue scanning. Our work demonstrates that our novel FF-ViT model, including the PAR-BMA components, outperforms SOTA models for this task. We provide formula derivations for our contributions and demonstrate an increase in performance for each contribution in an ablation study. Our performance evaluation verifies the accuracy of our network, its stability and generalizability on challenging datasets. As future work, we plan to combine our model with a longitudinal distance regression model to optimize both the position and orientation of the pCLE probe during robotic tissue scanning.

## Supplementary Information

Below is the link to the electronic supplementary material.

Supplementary file 1 (pdf 1799 KB)

## Footnotes

1 Code, dataset and supplemental materials are available at https://github.com/CVRS-Hamlyn/FF-ViT.
