# Supplementary material for: FF-ViT: probe orientation regression for robot-assisted endomicroscopy tissue scanning
Source: Int J Comput Assist Radiol Surg. 2024 Apr 10;19(6):1137–45. doi: 10.1007/s11548-024-03113-2 (PMC11582276; doi:10.1007/s11548-024-03113-2)
Supplement: Supplementary file 1 — (pdf 1799 KB) [file 11548_2024_3113_MOESM1_ESM.pdf]

Supplemental Material

Convergence and Stability Study

In the context of robotic scanning, the network's predictions are harnessed to guide the robot incrementally to the optimal scanning pose, taking steps sized $\theta_r = -\theta^2_{cls}$. To evaluate the network's convergence capabilities and stability in controlling the robot, we conducted two primary experiments: (1) Rotation signal analysis to confirm that the model accurately predicts the correct rotation angles for robotic control, and (2) Convergence evaluation to demonstrate the model's capacity to move the probe towards the optimal scanning position and maintain stability there [4]. Both of these experiments were carried out using the lens paper and cow heart test datasets.

For the rotation signal analysis, results are displayed in the first row of Fig 4. The figure's x and y axes represent the ground truth probe orientations and the predicted angles of rotation, respectively. The green line delineates the valid working range, while the red line indicates the ground truth, which serves as a comparison base for the predicted angles. When examining the lens paper dataset, it's clear that both FF-ViT and SFFC-Net deliver accurate predictions that closely align with the red line, while ResNet18's predictions significantly deviate from the red line, as emphasized by the purple circle. With respect to the cow heart dataset, most of FF-ViT's predictions adhere closely to the red line, whereas the predictions from SFFC-Net and ResNet18 significantly veer off from the red line, again highlighted by the purple circle. Consequently, the FF-ViT model demonstrates superior accuracy in predicting probe orientations across both datasets.

The assessment of convergence in the robotic probe manipulation is conducted using a K-step incremental analysis with image-feedback at each step, denoted as $k \in [1, \mathrm{K}]$. A pCLE image from the test dataset, associated with a known probe orientation θ_{gt}, is input into our trained network. At each iteration k, the inferred θ^k_r is utilized to update the pCLE probe's orientation according to the equation $\theta^{k+1}_{up} = \theta^k_{up} + \theta^k_r$, where $\theta^0_{up} = \theta_{gt}$. Our capability to carry out this procedure is greatly enhanced by the use of modified pivot point rotation, as described in section 3.1. Due to this modification, each video recording of the probe's rotation yields pCLE images targeting the same location. Consequently, we can generate the pCLE image acquired at the updated probe orientation θ^{k+1}_{up} via pixel-wise linear interpolation between the two nearest frames. This updated frame is then input into the trained network, and the process repeats until $k = K$.

The trends of probe-tissue orientations throughout the K-step incremental analysis are displayed in the second row of Fig. 4. The finely detailed solid lines in these figures track changes in probe orientations originating from a range of initial probe-tissue orientations. The accompanying grey shaded areas denote the standard deviation of the probe-tissue orientations at each step, given samples that share the same starting orientation. The red dotted line establishes the upper and lower limits of probe-tissue orientations when the process converges. As evidenced by the convergence figures within the lens paper dataset, all models converge successfully. However, the converged probe-tissue orientations generated by ResNet18 significantly shift towards the positive orientation. This marked shift, termed 'positive shifting', primarily results from the

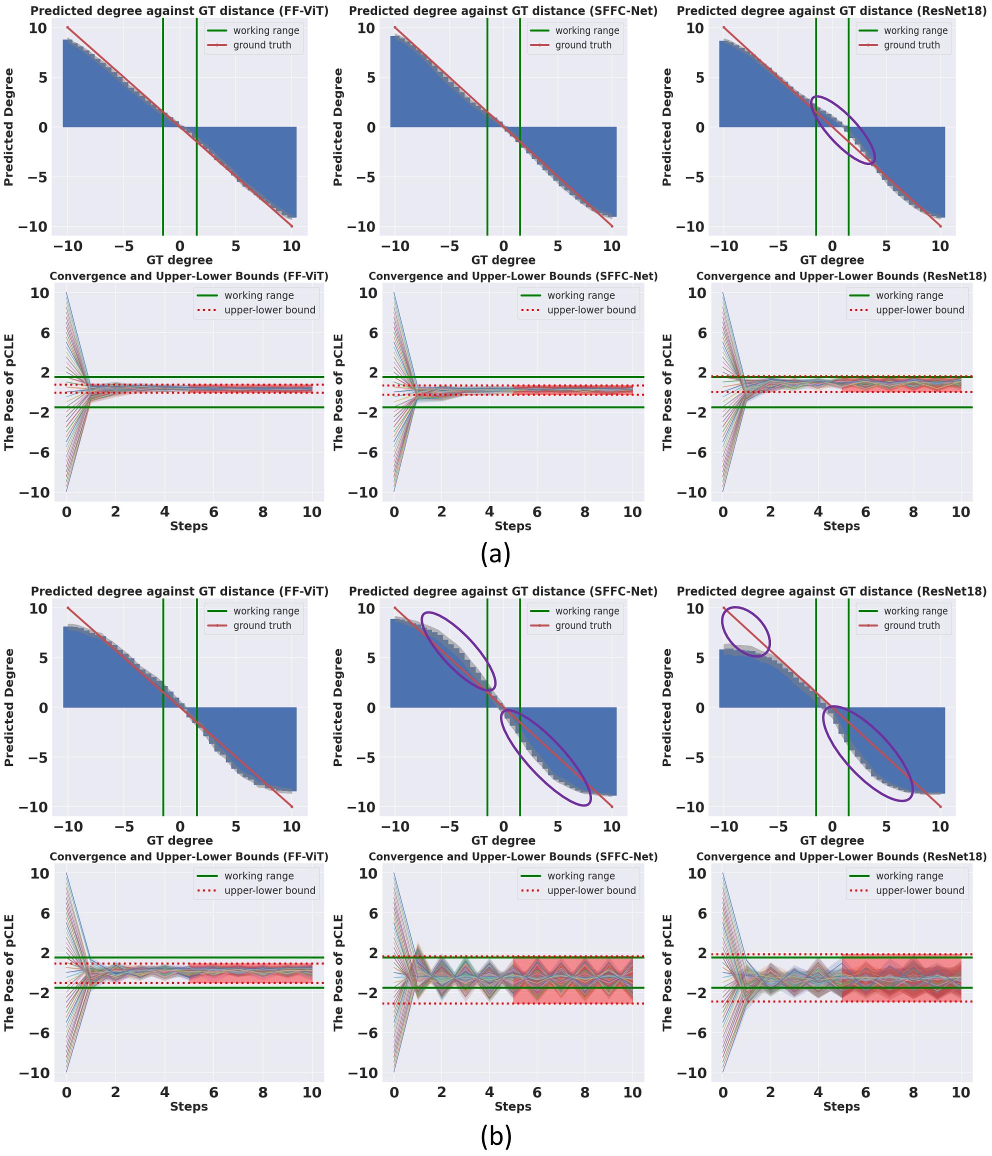

Fig. 4 (a) and (b): The convergence and stability study conducted on lens paper and cow heart dataset respectively.

significant deviation in predictions, as detailed in the rotation signal analysis. In the case of the cow heart dataset, FF-ViT successfully converges the probe-tissue orientation within the valid working range. Whilst, the converged orientations of SFFC-Net and ResNet18 demonstrate a substantial shift towards the negative orientation, termed 'negative shifting'. This can be explained by examining the purple circles in the first

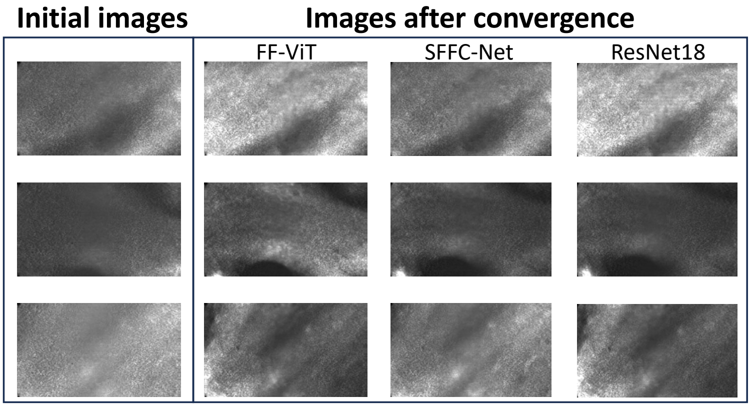

Fig. 5 Visualization of cow heart pCLE images acquired by models.

row of Fig. 4. Here, it's apparent that the negative-direction rotation signals predicted by both SFFC-Net and ResNet18 significantly exceed the ground truth. Furthermore, the positive-direction rotation signals predicted by ResNet18 fall notably below the ground truth, contributing to the observed negative shifting issue. These results underscore the importance of precise orientation prediction and the potential issues arising from inaccurate predictions, which can disrupt the stability and efficiency of the robotic probe manipulation, and consequently, the quality of the tissue scans. For visualization of results, some pCLE images acquired upon convergence are shown in Fig. 5.

Table 5 The convergence and stability study. The ✗ means that maximum outlier of probe-tissue orientation is out of the working space of robot.

	**Lens Paper**				
**Models**	**MAE**$_C$ (°) ↓	$\boldsymbol{\sigma_C}$ (°) ↓	**BW** (°) ↓	**outlier**$_{max}$ (°) ↓	**SIR** (%) ↑
AlexNet[27]	**1.07** ± 0.01	0.09	1.69	4.35	86.1%
VGG16[28]	1.44 ± 0.01	0.17	1.11	5.17	75.0%
ResNet18[23]	1.35 ± 0.01	0.14	1.56	**3.52**	62.6%
SFFC-Net[4]	1.23 ± 0.02	0.12	0.96	4.31	**90.6%**
FF-ViT	1.10 ± **0.00**	**0.07**	**0.79**	3.63	88.9%
	**Cow Heart**				
**Models**	**MAE**$_C$ (°) ↓	$\boldsymbol{\sigma_C}$ (°) ↓	**BW** (°) ↓	**outlier**$_{max}$ (°) ↓	**SIR** (%) ↑
AlexNet[27]	2.28 ± 0.03	1.44	3.97	8.37	68.0%
VGG16[28]	3.51 ± 0.15	2.50	7.69	✗	65.0%
ResNet18[23]	3.19 ± 0.18	1.04	4.71	✗	**83.9%**
SFFC-Net[4]	2.46 ± **0.01**	1.49	4.71	8.13	77.5%
FF-ViT	**1.39** ± 0.07	**0.46**	**1.77**	**4.59**	73.6%

The convergence study uses five evaluation metrics to assess model performance, as detailed in Table 5. These include Mean Absolute Error at Convergence ($\mathbf{MAE}_C$), standard deviation at convergence ($\boldsymbol{\sigma}_C$), Bound Width (**BW**), Maximum Outlier ($\mathbf{outlier}_{max}$), and Samples Inside Range percentage (**SIR**). The $\mathbf{MAE}_C$ and $\boldsymbol{\sigma}_C$ measure the mean absolute error between the updated probe pose and the tissue surface normal, and the variation in predicted orientations upon convergence, respectively. The **BW** reflects the width of the upper-lower bound, while $\mathbf{outlier}_{max}$ and **SIR** capture the greatest deviation in probe-tissue orientations and the proportion of samples within the valid working range at convergence.

As per Table 5, FF-ViT exhibits the lowest **BW** and $\boldsymbol{\sigma}_C$ across both datasets, illustrating its ability to guide the robot to the optimal pose in a stable manner. FF-ViT also records the second-lowest $\mathbf{outlier}_{max}$ (only 0.11° higher than the lowest) in the lens paper dataset, and the lowest in the cow heart dataset, indicating safer feedback signals for robotic control. Although FF-ViT's **SIR** trails SFFC-Net and ResNet18, its superior performance across the remaining four metrics shows its overall effectiveness. In summary, the convergence study establishes that FF-ViT can generate the most stable and safest rotation signals for robotic control. Additionally, FF-ViT's impressive performance across both datasets attests to its superior generalizability compared to other models.

Blur Metrics for Image Quality Estimation

In this section, 5 popular blur metrics are analysed to be used later in our performance evaluation study.

Mean of Intensity (MoI) According to the confocal property of the pCLE probe, the incoming laser beams are blocked (*i.e.* the detected photons are significantly reduced) when the probe is out of focus (outside the working distance). Instead of evaluating the clarity of the pCLE images, counting the number of detected photons (intensity) can also assess the data quality. Therefore, the mean of intensity has been proposed by [4] and it can be expressed as,

$$\mathrm{MoI} = \frac{1}{N}\sum I, \tag{7}$$

where, $I \in \mathbb{R}^{H\times W}$ is the input image and N is the number of pixels in the image I.

Non-reference Blur Metric (NRBM) This blur metric has been proposed by [18]. At first, the averaging kernel is used to blur the original image,

$$\mathbf{B}_h = \frac{1}{9}\times[111111111], \mathbf{B}_v = \mathbf{B}_h^T, \tag{8}$$

$$I_h^B = conv(I, \mathbf{B}_h), I_v^B = conv(I, \mathbf{B}_v), \tag{9}$$

where, $conv(\cdot)$ is the convolution function. Then, the variance along the horizontal and vertical direction, between adjacent pixels of the original image (D_h and D_v) and

the blurred image (D_h^B and D_v^B) are computed as:

$$\begin{aligned} D_h &= \left| I_{h,2} - I_{h,1} \right|, D_v = \left| I_{v,2} - I_{v,1} \right|, \\ D_h^B &= \left| I_{h,2}^B - I_{h,1}^B \right|, D_v^B = \left| I_{v,2}^B - I_{v,1}^B \right|, \end{aligned} \tag{10}$$

where, $\left\{ I, I^B \right\}_{\{h,v\},\{1,2\}}$ are sub-matrices of I, expressed as,

$$\begin{aligned} I_{h,1} &= (I_{i,j}), I_{h,1}^B = (I_{i,j}^B), 1 \le i \le H, 1 \le j \le W-1, \\ I_{h,2} &= (I_{i,j}), I_{h,2}^B = (I_{i,j}^B), 1 \le i \le H, 2 \le j \le W, \\ I_{v,1} &= (I_{i,j}), I_{h,1}^B = (I_{i,j}^B), 1 \le i \le H-1, 1 \le j \le W, \\ I_{v,2} &= (I_{i,j}), I_{v,2}^B = (I_{i,j}^B), 2 \le i \le H, 1 \le j \le W. \end{aligned} \tag{11}$$

Finally, the difference between the original image and the blurred image along the horizontal and vertical direction can be computed as:

$$\begin{aligned} \mathbb{D}_h &= \sum (\max(0, D_h - D_h^B)) \\ \mathbb{D}_v &= \sum (\max(0, D_v - D_v^B)) \end{aligned} \tag{12}$$

848

If the original image is clear, the difference will be large, and vice versa. Therefore, the maximum value between $\mathbb{D}_h$ and $\mathbb{D}_v$ is used to describe the pCLE image clarity as:

$$\text{NRBM} = \max(\mathbb{D}_h, \mathbb{D}_v) \tag{13}$$

High-pass Fast Fourier Transform (H-FFT) The fast Fourier transform $f_{fft}(\cdot)$ is used to convert images from the spatial domain to the frequency domain. Each element at frequency domain represents 2D wave with a particular frequency at spatial domain. After shifting $f_{shift}(\cdot)$, the elements close to the centre of the frequency domain represent low frequencies, and vice versa. Since the high frequency signal is correlated to the clarity of an image, the high-pass filter $f_{hp}(\cdot)$ can be used to only preserve the elements with high frequencies:

$$I_h = f_{hp}\left(f_{shift}\left(f_{fft}(I)\right)\right) \tag{14}$$

Then, the inverse fast Fourier transform $f_{ifft}(\cdot)$ is applied to transform these high frequency signals into the spatial domain. The summation of these signals represents image clarity as:

$$\text{FFT} = \sum f_{ifft}(I_h) \tag{15}$$

Variance of Laplacian (LAPV) The *Laplacian* kernel is a convolutional kernel commonly used in edge detection. It is aimed to compute the second order derivative

of an image and it is defined as:

$$\mathbf{L} = \begin{bmatrix} 0 & 1 & 0 \\ 1 & -4 & 1 \\ 0 & 1 & 0 \end{bmatrix} \tag{16}$$

The clearer the image, the greater the variance of its convolution result. Hence, the clarity can be quantified as:

$$\text{LAPV} = \frac{\sqrt{(conv(I, \mathbf{L}) - (\sum conv(I, \mathbf{L}))\,/N)^2}}{N} \tag{17}$$

Gaussian derivative (GDER) Different from the *Gaussian smoothing* kernel, the *Gaussian derivative* kernel is the first order derivative of the 2D *Gaussian smoothing* kernel, which is widely used for edge detection. The *Gaussian smoothing* kernel and the *Gaussian derivative* kernel are mathematically defined as:

$$\mathbf{G} = \frac{1}{2\sigma\pi} \cdot e^{\frac{-(x^2+y^2)}{2\sigma^2}} \tag{18}$$

$$\mathbf{G'_x} = \frac{-x \cdot \mathbf{G}}{\sigma^2 \cdot |\mathbf{G}|}, \mathbf{G'_y} = \frac{-y \cdot \mathbf{G}}{\sigma^2 \cdot |\mathbf{G}|} \tag{19}$$

where, $\mathbf{G}'_x$ and $\mathbf{G}'_y$ are *Gaussian derivative* kernels along the x and y direction, respectively. The clarity of an image is positively correlated with the sharpness of the image edges. Therefore the clarity can be quantified as:

$$\text{GDER} = \frac{1}{N} \sum (conv(I, \mathbf{G}'_x)^2 + conv(I, \mathbf{G}'_y)^2) \tag{20}$$
